# Comparison of the Efficacy of Intralesional Tranexamic Acid Versus Topical 4% Hydroquinone in Treating Melasma

**DOI:** 10.7759/cureus.28547

**Published:** 2022-08-29

**Authors:** Shigref Mushtaq, Syeda Sibgha Naz, Muhammad Rizwan, Nudrat Jehangir Khan, Obed Ullah, Anjum Muhammad

**Affiliations:** 1 Department of Dermatology, Pak Emirates Military Hospital, Rawalpindi, PAK

**Keywords:** melasma, hydroquinone, hyperpigmentation, intradermal, intralesional tranexamic acid

## Abstract

Objectives: To compare the efficacy of intralesional tranexamic acid and topical 4% hydroquinone in the treatment of melasma.

Study design: This comparative prospective study was conducted at the Dermatology Department, Pak Emirates Military Hospital Rawalpindi, Pakistan from October 16, 2018 to April 16, 2019.

Methodology: A total of 290 patients with melasma, 18 to 50 years of age, were included in this study. Patients with a history of discoid lupus erythematosus, pregnancy, lactation, anemia, and oral contraceptives or hormone replacement therapy were excluded from the study. Randomization was 1:1 for groups A and B, i.e., each upcoming patient was included in the next group. This randomization was supervised by another clinician. One hundred forty-five patients were placed in group A (intralesional tranexamic acid), while 145 were enrolled in group B (topical 4% hydroquinone). Follow-up was done at four weekly intervals for 12 weeks. After 12 weeks, the final response was assessed.

Results: In group A, the average age was 33.74 ± 6.67 years, while in group B it was 32.08 ± 6.08 years. Among the entire patients, the majority of the patients, 207 (71.38%), were in the age range of 18 to 35 years. Intralesional tranexamic acid was efficacious in 64 (44.14%) patients, while 47 (32.41%) of group B (topical 4% hydroquinone) showed complete improvement (p-value = 0.040).

Conclusion: This study concluded that using intralesional tranexamic acid is more effective in treating melasma than topical 4% hydroquinone.

## Introduction

Melasma is defined as an acquired pigmentation disorder that affects photo-exposed areas of the body, most commonly the face, neck, and forearms [1]. It is very common in Southeast Asia, where it affects 40% of females and 20% of males. Melasma accounts for half of all aesthetic consultations in Asia. Females have a higher prevalence than males, with a female-to-male ratio of about 4:1. Women who have had many pregnancies have a greater rate (51%) than unmarried women (25%) or women who have never had a pregnancy (24%) [2].

Despite advancements in dermatological therapies, the treatment of melasma is challenging. Melasma can be treated with a variety of approaches, such as sun protection, avoidance of triggering factors, topical depigmenting agents, oral medications, laser therapy, dermabrasion, and chemical peels, with varying degrees of success [2]. A number of new pigment-producing mechanisms are being investigated as potential targets for topical treatment. Topical drugs include hydroquinone (HQ), tretinoin, azelaic acid (AA), kojic acid, corticosteroids, and their different combinations, chemical peels like glycolic acid (GA), salicylic acid, lactic acid, trichloroacetic acid, phenol, and laser therapy are all used to treat melasma [3].

Hydroquinone (HQ), a phenolic molecule, inhibits tyrosinase, which prevents 3,4-dihydroxyphenylalanine (DOPA) from being converted to melanin and lowers dyschromia by downregulating melanocytes, preventing melanosome formation, and reducing melanin transfer to keratinocytes. It's most usually given at a 4% concentration, although it's also available in 10% and 2% concentrations. It works well when applied on the whole face once daily because a localized application can induce bull's-eye patches of discoloration.

Trans-4-(Aminomethyl)cyclohexane carboxylic acid (TNA) prevents fibrinolysis by inhibiting plasmin and hence reduces blood loss [4]. It is a synthetic lysine derivative that blocks plasminogen's lysine binding sites, preventing it from converting to plasmin. It is assumed that plasminogen affects keratinocytes since it is found in human epidermal cells and cultured keratinocytes. Tranexamic acid 250 mg was given twice daily for a duration of three months and it significantly reduced the melasma area and severity index (MASI) score [5]. It has seldom been linked to thromboembolism, pulmonary embolism, and myocardial infarction [6].

Both intralesional tranexamic acid and hydroquinone 4% have their individual efficacy as mentioned above [7,8]. However, their relative efficacy has not been tested, and there are no published data, especially in our South Asian population pertaining to this subject. Moreover, the results of international studies cannot be generalized to our local population due to differences in skin color and socio-economic conditions. Melasma is a common yet difficult to cure skin disorder that recurs frequently. It has a huge psychological impact, so I have planned to compare the efficacy of intralesional tranexamic acid and topical 4% hydroquinone in treating melasma. The result of our study will help to select a better treatment modality for patients suffering from melasma.

## Materials and methods

This comparative prospective study was conducted at the Dermatology Department, Pak Emirates Military Hospital Rawalpindi, Pakistan from October 16, 2018 to April 16, 2019. A sample of 290 women with melasma for the last six months with a MASI score ≥ 10 was selected through a consecutive non-probability sampling technique.

Inclusion criterion

Inclusion criteria were both married and unmarried patients of age 18-50 years having melasma for six months and had not received any treatment for melasma in the last three months.

Exclusion criteria

Patients with a history of discoid lupus erythematosus, lichen planus on their medical records, pregnancy, lactating mothers, anemia (Hb <10), and patients on oral contraceptive pills or hormonal therapy were excluded.

Data collection

The patients' consent was obtained after receiving clearance from the Institutional Ethical Review Committee of Pak Emirates Military Hospital, Rawalpindi having a reference number of A/28. Baseline demographics (age, length of complaint, marital status, melasma pattern, and baseline MASI score) were collected at the start of the study. By using a block design, we were able to randomize the data. Randomization was 1:1 for group A and group B, i.e., each upcoming patient was included in the next group; this randomization was supervised by another clinician. The sample size for group A (intralesional tranexamic acid) was 145, while the sample size for group B (topical 4% hydroquinone) was 145.

Patients in group A received weekly intradermal injections of 0.5 ml tranexamic acid in 0.5 ml of normal saline (50 mg/ml) into the melasma lesion at 1 cm intervals using a sterile insulin syringe. Patients in group B were given a topical hydroquinone 4% cream to apply to the relevant area at night for a period of 12 weeks. Follow-up was done at four weekly intervals for 12 weeks. After 12 weeks, the final response was assessed. All patients were called for follow-up and efficacy was evaluated on the basis of a fall in MASI score from baseline. Patients who missed follow-ups were contacted through their contact numbers and were reminded to come for their follow-up visit.

Data analysis

**Figure 1:**
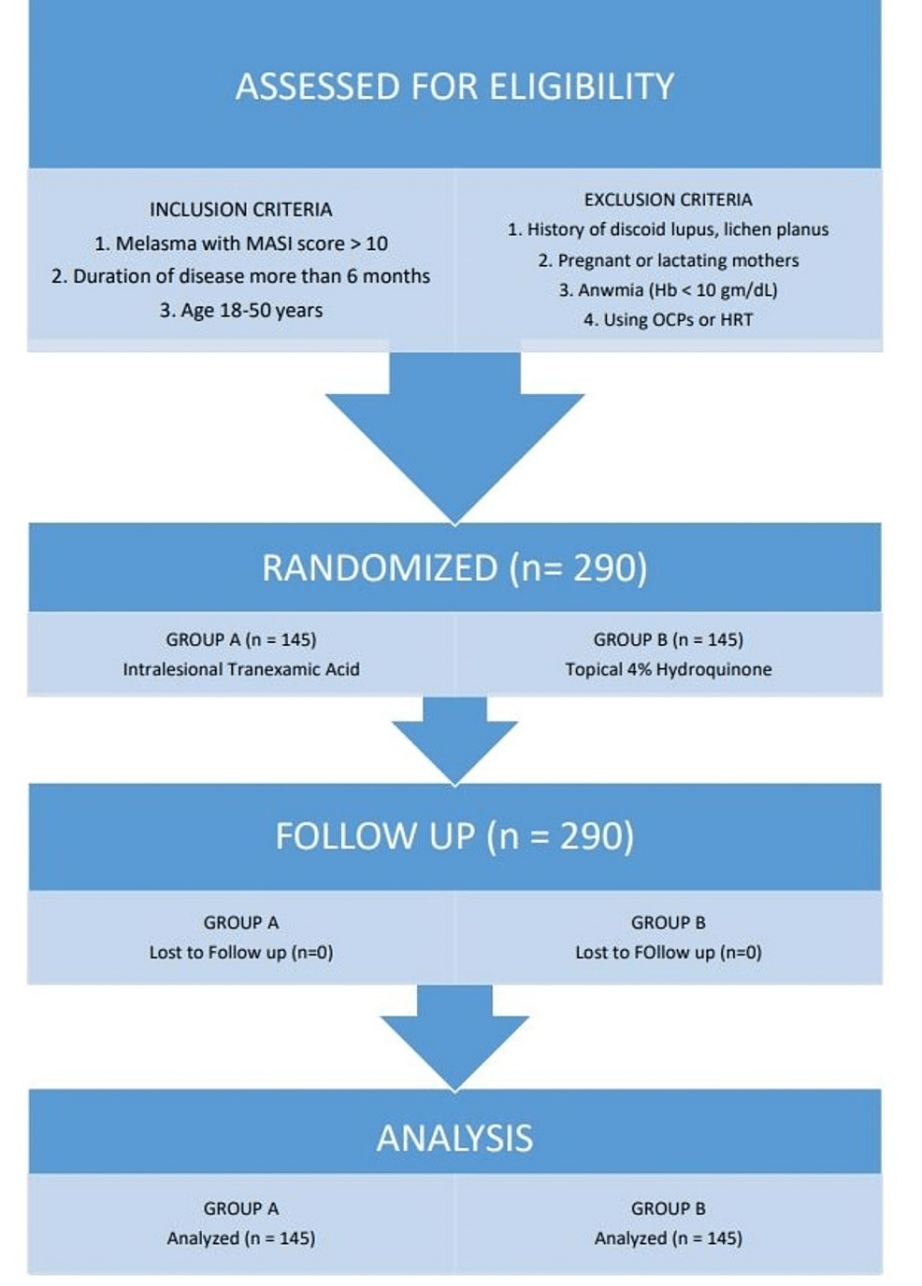
The flow diagram showing the overall study design with inclusion and exclusion criteria. MASI: melasma area and severity index, OCPs: oral contraceptive pills, and HRT: hormonal therapy.

A statistical analysis program was used to analyze the data (IBM-SPSS version 23, IBM Corporation, New York). For quantitative data, the mean and standard deviation were used. For qualitative variables, the frequency and percentage were calculated. The Chi-square test was used to compare efficacy in both groups, with a significance level of p ≤ 0.05. The flow diagram shows the overall study design (Figure 1).

## Results

The participants in this study ranged in age from 18 to 50 years old, with a mean age of 32.88 ± 6.23 years. Patients in group A were 33.74 ± 6.67 years old, whereas those in group B were 32.08 ± 6.08 years old. The majority of the patients, 207 (71.38%), were between the ages of 18 and 35, as shown in Table 1.

**Table 1:** Age distribution for both groups (n = 290).

Age (years)	Group A (n = 145)		Group B (n = 145)		Total (n = 290)	
	No. of patients	% Age	No. of patients	% Age	No. of patients	% Age
18-35	100	68.97	107	73.79	207	71.38
36-50	45	31.03	38	26.21	83	28.62
Mean ± SD	33.74 ± 6.67		32.08 ± 6.08		32.88 ± 6.23	

A total of 207 (71.38%) patients were females and 83 (28.62%) were males, with a female to male ratio of 2.5:1. The mean duration of disease was 9.77 ± 2.13 months (Table 2). Stratification of efficacy with respect to melasma pattern is shown in Table 3.

**Table 2:** Percentage of patients according to the duration of disease (n = 290).

Duration of disease (months)	Group A (n = 145)		Group B (n = 145)		Total (n = 290)	
	No. of patients	% Age	No. of patients	% Age	No. of patients	% Age
6-9 months	81	55.86	80	55.17	161	55.52
>9 months	64	44.14	65	44.83	129	44.48
Mean ± SD	9.74 ± 2.25		9.85 ± 2.00		9.77 ± 2.13	

**Table 3:** Stratification of efficacy with respect to melasma pattern.

Melasma pattern	Group A (n = 145)		Group B (n = 145)		P-value
	Efficacy		Efficacy		
	Yes	No	Yes	No	
Centro-facial	28	40	20	43	0.354
Malar	20	31	21	39	0.647
Mandibular	16	10	06	16	0.018

Efficacy of group A (intralesional tranexamic acid) was seen in 64 (44.14%) patients, while group B (topical 4% hydroquinone) was seen in 47 (32.41%) patients, as shown in Table 4 (p-value = 0.040).

**Table 4:** Comparison of the efficacy of intralesional tranexamic acid and topical 4% hydroquinone in treating melasma.

Efficacy	Group A (n = 145)	Group B (n = 145)	P-value
Yes	64 (44.14%)	47 (32.41%)	0.040
No	81 (55.86%)	98 (67.59%)	

## Discussion

Melasma is a common dermatitis characterized by brown-colored hyperpigmented areas, found mainly on sun-exposed parts of the face and around the neck. The pathology of melasma is still unclear. However, the causes are likely ultraviolet (UV) exposure, hormonal imbalance, thyroid disease, contraceptive pills, irrational use of cosmetics, and phototoxic drugs. The usual treatment modalities include measures to nullify the effects of the above causes and the use of sunscreens, depigmentation agents, along with a combination of laser treatments. Earlier studies were carried out on oral, topical, and intravenous doses of tranexamic acid and analyzed the efficacy of tranexamic acid in various routes of administration with different dosages [9,10]. Recent advancements in the treatment of melasma with tranexamic acid promise favorable results in UV-induced melasma [10].

We have conducted this study to compare the efficacy of intralesional tranexamic acid and topical 4% hydroquinone in treating melasma. The mean age of patients in this study was 32.88 ± 6.23 years, which was very much comparable with Ejaz et al. [11] who had a mean age of 32 years but higher than Bari et al. [12] and Ethawi and Sidiq [13] who had a mean age of 23 years. On the other hand, Lee et al. [14] found a much larger mean age, i.e., 41 years, in comparison to our research. In our study, a total of 207 (71.38%) patients were females and 83 (28.62%) were males, with a female to male ratio of 2.5:1. Many previous studies have also shown female predominance as observed in our study [11-14].

The majority of patients in our study were between the ages of 20 and 40. Similar findings were also observed by Al-Hamdi et al. [15]. It affects people of all ethnicities. However, it is more common in people of darker complexions than in lighter skin, notably in Hispanic and Asian people [8].

In our study, the efficacy of group A (intralesional tranexamic acid) was seen in 64 (44.14%) patients, while in group B (topical 4% hydroquinone) it was seen in 47 (32.41%) patients. A study by Rassai et al. has shown that the efficacy of hydroquinone (4%) was 36.7% [8,16] and 31% [17] in the treatment of melasma. In another study, it was revealed that 38% of patients showed complete recovery after using hydroquinone, while 4% of patients showed a very good response [18].

Xu et al. in 2017, found that penetrating the epidermal barrier with microneedles and tranexamic acid solution improved melasma significantly [19], which was in accord with Lee et al., although the results were achieved with micro-needling followed by the application of the tranexamic acid solution on diseased areas to facilitate epidermal absorption. Self-controlled split-face research on 28 women, performed weekly for 12 weeks, came to similar conclusions about tranexamic acid efficacy and safety. According to the findings, 25 patients treated with micro-needling plus tranexamic acid improved by more than 25%, while only 10% improved on the control side of the face, which was treated with only 0.5% topical tranexamic acid [19].

Following tranexamic acid administration to neonatal foreskin-cultured melanocytes before ultraviolet B (UVB) irradiation, Seo et al. observed a substantial suppression of melanocyte proliferation. They also found lower tyrosinase activity, lower expression of tyrosine-related proteins 1 and 2, and lower melanin concentration. However, there was no change in the length of dendrites of melanocytes [20]. A study by Hongjin and Xihui concluded that the duration of treatment was more important than the dose of tranexamic acid [21].

Na et al. reported a decline in epidermal melanin pigmentation, vessel numbers, and mast cell counts after histological examination [22]. There was also a considerable decrease in the lesional melanin index. Following weekly intradermal injections of tranexamic acid (4 mg/ml), each site received 0.05 ml of tranexamic acid, which was 1 cm apart. 

Wu et al. observed a notable response in 96% of the 74 women who received tablet tranexamic acid in the dose of 250 mg twice daily for a period of six months for melasma. About 5.4% of the study population had some sort of gastrointestinal discomfort, and 8.1% developed hypomenorrhea. This indicated different pathogenetic actions of tranexamic acid in melasma. Improvement started to appear after four to eight weeks [23].

In a study by Kim et al., 2% topical tranexamic acid was applied to 23 melasma patients for 12 weeks, and 22 out of 23 participants saw a significant improvement in their modified MASI score. The melanin concentration of the epidermis was found to be significantly reduced by Fontana-Masson staining. Endothelin-1 (ET-1) was also found to be downregulated [24]. In a large study by Lee et al. in 2016, oral tranexamic acid was given to 561 patients with melasma. About 89.7% showed improvement, while 10% did not show any improvement. Among those cases who responded, improvement was seen in two months. A relapse rate of 27.2% was seen. One patient developed deep vein thrombosis (DVT) and required discontinuation of therapy. She was later found to be a case of familial protein S deficiency [25].

A recent split-face study by Saki et al. comparing monthly intradermal tranexamic acid and daily 2% hydroquinone cream showed a marked reduction in melanin value on each side. Monthly tranexamic acid showed better improvement at the end of four weeks, but the results were comparable at the end of 20 weeks [26]. Another recent study by Atefi et al. compared the 5% tranexamic acid cream to the 2% hydroquinone cream and found significant improvements in MASI scores in both groups but no significant differences between them. A higher level of patient satisfaction was seen in the tranexamic acid group (33.3% vs. 6.7% in the hydroquinone group). The hydroquinone group also had a greater rate of side effects such as skin irritation and erythema (10% vs. no major side effects in the tranexamic acid group) [27].

## Conclusions

Our study, being a single center work, with 290 patients, showed that melasma is a very common disease in younger age groups and centro-facial melasma is the most common pattern that we observe in our patients, followed by malar and mandibular patterns. Moreover, melasma is observed more in married male and female patients.

This study revealed that intralesional tranexamic acid is the more effective and safe therapeutic modality in treating melasma than topical 4% hydroquinone. As a result, we recommend that intralesional tranexamic acid be utilized in the treatment of melasma to improve these patients' social lives, emotional well-being, and leisure activities.
